# Abscess of Anus in Typhoid Fever

**Published:** 1876-04

**Authors:** 


Abscess of the Anus in Typhoid Fever.—Chalot (*Montpellier Med.*, 1875, No. 5, *Centralbl. f. Chir.*, 1876, No. 5) gives the case of a soldier, 23 years of age, suffering from a severe attack of typhoid fever, in the course of which an abscess made its appearance in the region of the rectum, which broke spontaneously, and resulted in a fistula. In giving the case, Chalot takes the opportunity to record the frequency of this complication of typhoid fever, and recommends the use of the elastic ligature.

